# Enhancing Optical Forces in InP-Based Waveguides

**DOI:** 10.1038/s41598-017-03409-1

**Published:** 2017-06-08

**Authors:** Mohammad Esmail Aryaee Panah, Elizaveta S. Semenova, Andrei V. Lavrinenko

**Affiliations:** Technical University of Denmark, Department of Photonics Engineering, Ørsteds Plads, Building 343, DK-2800 Kgs, Lyngby, Denmark

## Abstract

Cantilever sensors are among the most important microelectromechanical systems (MEMS), which are usually actuated by electrostatic forces or piezoelectric elements. Although well-developed microfabrication technology has made silicon the prevailing material for MEMS, unique properties of other materials are overlooked in this context. Here we investigate optically induced forces exerted upon a semi-insulating InP waveguide suspended above a highly doped InP:Si substrate, in three different regimes: the epsilon-near-zero (ENZ), with excitation of surface plasmon polaritons (SPPs) and phonons excitation. An order of magnitude amplification of the force is observed when light is coupled to SPPs, and three orders of magnitude amplification is achieved in the phonon excitation regime. In the ENZ regime, the force is found to be repulsive and higher than that in a waveguide suspended above a dielectric substrate. Low losses in InP:Si result in a big propagation length. The induced deflection can be detected by measuring the phase change of the light when passing through the waveguide, which enables all-optical functioning, and paves the way towards integration and miniaturization of micro-cantilevers. In addition, tunability of the ENZ and the SPP excitation wavelength ranges, via adjusting the carrier concentration, provides an extra degree of freedom for designing MEMS devices.

## Introduction

MEMS are microscopic devices with moving parts which deflect or vibrate upon applying a force. They have been studied during the last three decades and are currently utilized for commercial applications such as controlling fluid jets in inkjet printers, acceleration sensors for deploying car airbags^1^, fine-pointing mirrors for intersatellite optical links^2^ and tunable vertical-cavity surface-emitting lasers (VCSELs)^3^. Among various MEMS devices, cantilever sensors have attracted considerable attention due to their applications in ultra-sensitive mass sensing^4, 5^ and label-free detection of biological molecules^6^. Adsorption of molecules on the surface of a deformable micro-cantilever changes its mass and stiffness and consequently its mechanical resonance frequency. Selective detection of different molecules can be realized by functionalizing the surface of a cantilever with specific receptors. MEMS systems are evolving with improvements in fabrication processes in order to shrink the size, reduce the mass and increase the resonance frequency. Most of the currently available MEMS devices are actuated by electrostatic forces, piezoelectric elements or bilayers of different thermal expansions. The induced motion in micro-cantilevers is usually detected by optical interference or deflection of a laser beam reflected from their surface^1^.

During the past decade optically induced forces which arise due to coupling between the evanescent tail of a guided wave in a waveguide and a substrate or another waveguide have become an independent subject of research. In 2005 Povinelli *et al*. theoretically investigated the evanescent wave-bonding between dielectric optical waveguides. They reported forces in the range of piconewtons, which are enough to deflect a 30 µm long waveguide by approximately 20 nm^7^. Afterwards a series of papers was published which experimentally proved this concept. Li *et al*. in 2008 detected the displacement in a Si waveguide above a SiO_2_ substrate stimulated by the optical forces. They monitored the phase shift of light when it passes through a waveguide to detect the deflection^8^. The same group in 2009 published a paper in which they investigated the deflection induced in two evanescently coupled micro-cantilevers^9^. They used the change in the transmission through the waveguide system upon deflection of the cantilevers for characterization of the deflection. When the dielectric substrate is replaced with a metal one, light can couple to SPPs on the substrate surface, resulting in hybrid plasmonic modes^10^. The force between the waveguide and the substrate (in this case attractive) will be amplified due to the electric field enhancement. The effect of coupling of the light to surface phonon polaritons on optical forces in two adjacent SiC waveguides is theoretically investigated by Li *et al*., and an order of magnitude amplification of the force, in comparison to hybrid plasmonic waveguides, is reported^11^.

In this paper we study the optically induced forces exerted on an InP waveguide, which lies above an InP:Si substrate. The choice of material is caused by two reasons. First, the whole system consists of one material, which can be epitaxially grown and processed to deliver desired properties and functionalities. Second, it allows us to monitor three different scenarios of optomechanical interactions on the same material platform, namely the ENZ, SPP resonance, and the phonon resonance regimes. We address them by sweeping different frequency ranges. It is shown that the induced attractive forces can be drastically increased upon coupling to the surface plasmons or phonons. The working wavelengths for the ENZ regime and SPP coupling regime can be effectively tuned by changing the carrier concentration of InP^12^. Deflection of the waveguide can be determined by measuring the phase change of light propagating through the deflected waveguide similar to reported in ref. 8. Simultaneous on-chip optical actuation and detection paves the way towards miniaturization and integration of cantilever sensing devices, which are of great interest for industrial applications.

## Methods

**Figure 1:**
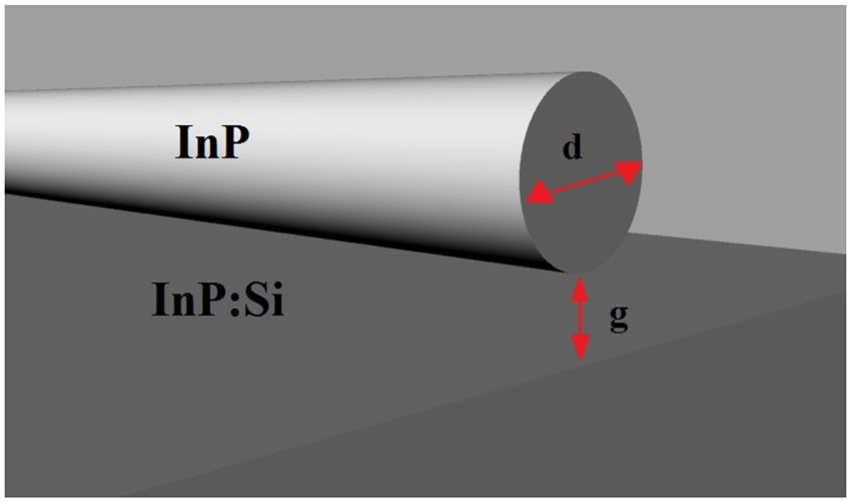
Schematic of the waveguide above a substrate.

Consider the waveguide in Fig. 1, separated from the substrate by gap *g*. Coupling between the evanescent tail of the mode guided inside the waveguide, and the substrate results in an optically induced force given by ref. 7

1 $$F=\frac{1}{{n}_{eff}}\frac{\partial {n}_{eff}}{\partial g}U,$$

where *n*_*eff*_ is the effective mode index, and *U* is the total field energy given by

2 $$U=PL{n}_{g}/c$$

with *P* the total optical power, *L* the length of the waveguide, *c* the speed of light and *n*_*g*_ the group index defined as

3 $${n}_{g}=\frac{c}{{v}_{g}}=c\frac{\partial k}{\partial \omega }=\frac{\partial }{\partial \omega }(\omega \,{n}_{eff}(\omega ))={n}_{eff}(\omega )+\omega \frac{\partial {n}_{eff}}{\partial \omega }.$$

**Figure 2:**
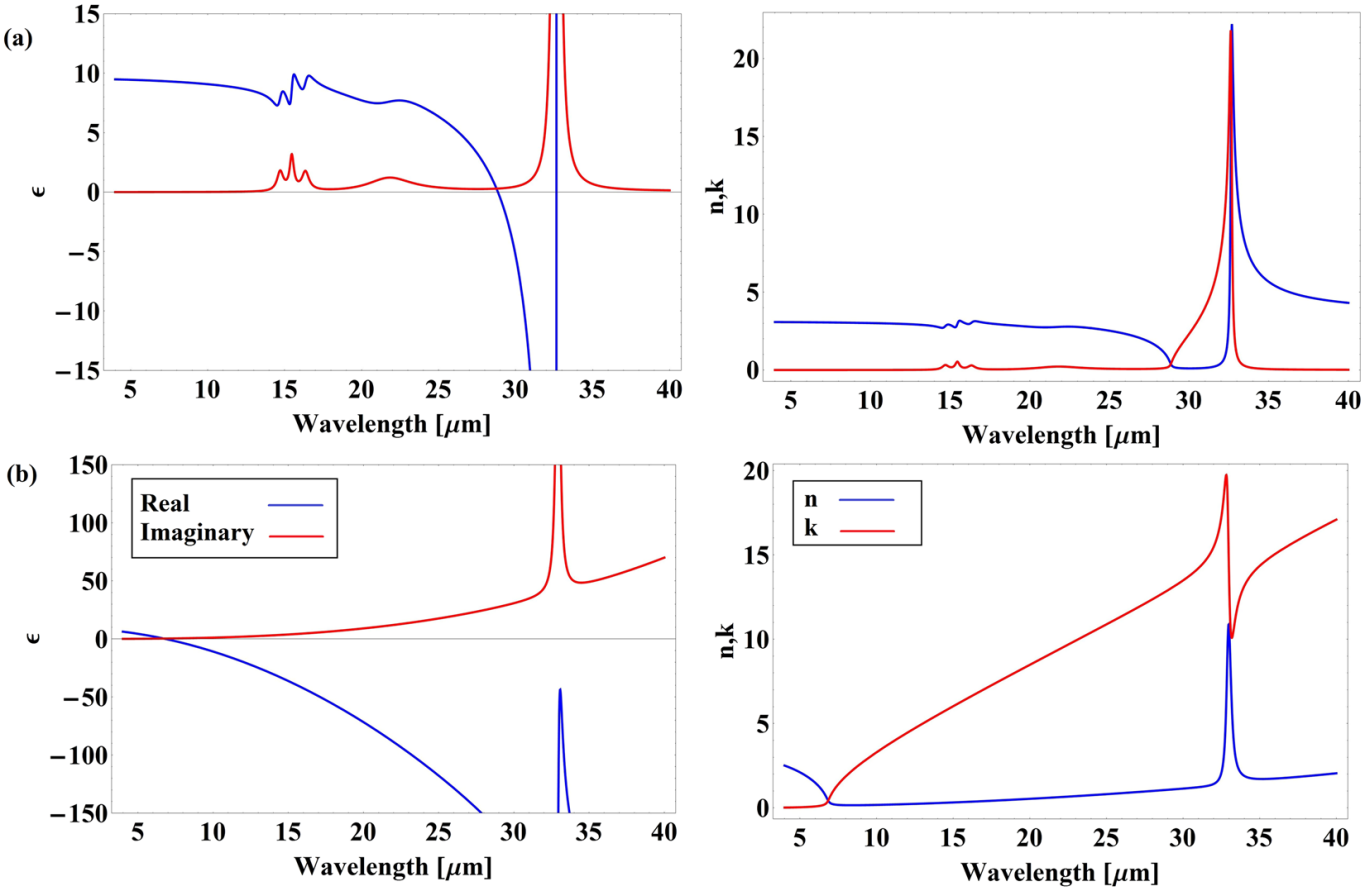
Real and imaginary parts of the permittivities and refractive indices of (**a**) semi-insulating InP and (**b**) InP:Si.

Figure 2 shows the experimentally determined^12^ real and imaginary parts of the permittivities and refractive indices of highly doped InP:Si (free carrier concentration 3.09 × 10^19^ cm^−3^) and semi-insulating InP. Using these values *n*_*eff*_ was calculated by the finite element method (COMSOL Multiphysics 5.0), for a range of gap sizes and input wavelengths. Afterwards, $\frac{\partial {n}_{eff}}{\partial g}$ and $\frac{\partial {n}_{eff}}{\partial \omega }$ were calculated numerically and used in (1) to calculate the force.

## Results

### Dielectric substrate

**Figure 3:**
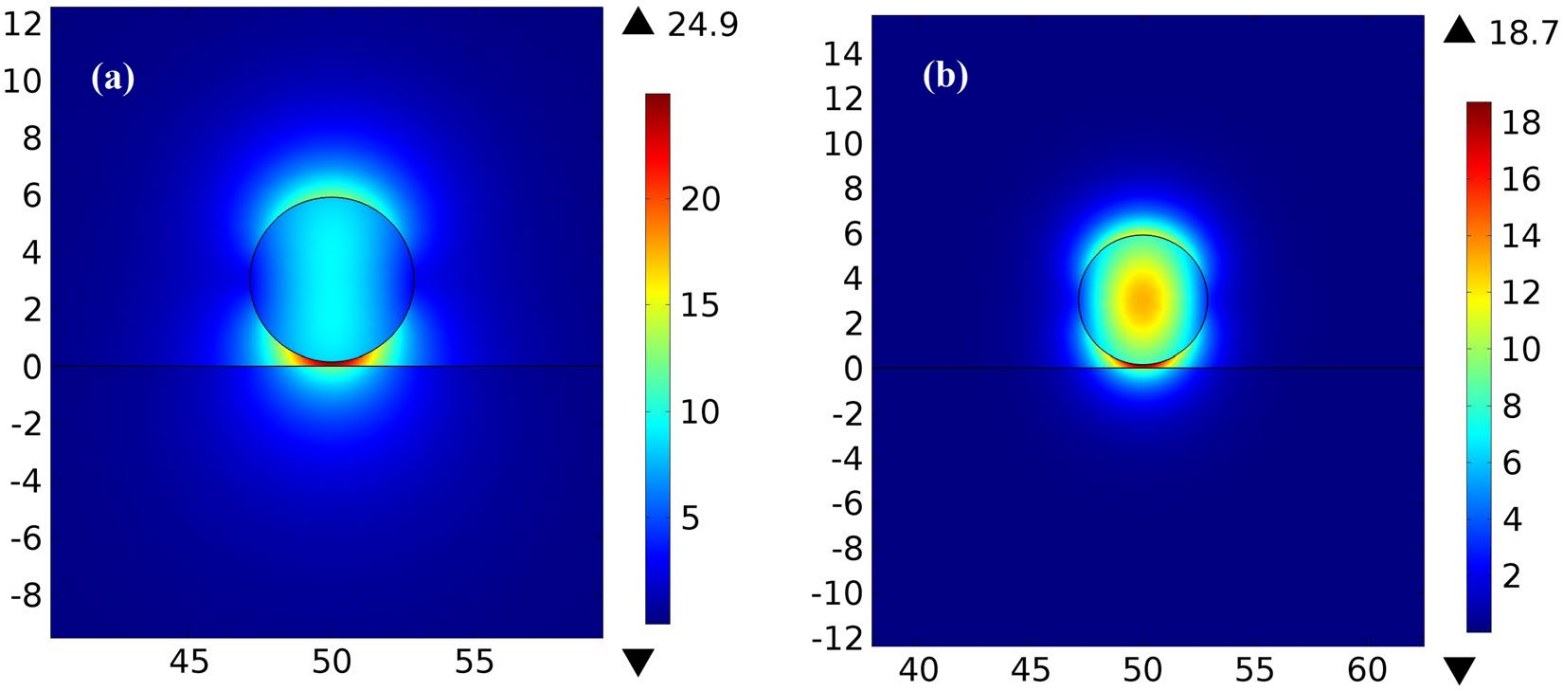
Simulated electric field [V/m] for (**a**) semi-insulating InP waveguide above a glass substrate (**b**) Si waveguide above a glass substrate at *λ*_0_ = 20 µm. Spatial dimensions are in µm.

**Figure 4:**
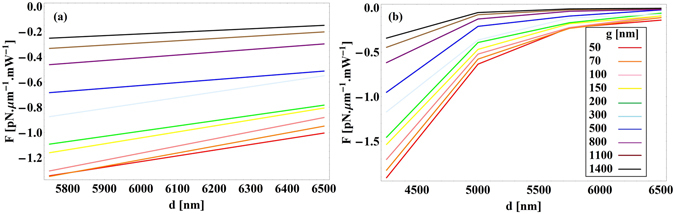
Optical force versus waveguide’s diameter for different gap sizes for (**a**) semi-insulating InP waveguide above a glass substrate and (**b**) Si waveguide above a glass substrate at *λ*_0_ = 20 µm.

**Figure 5:**
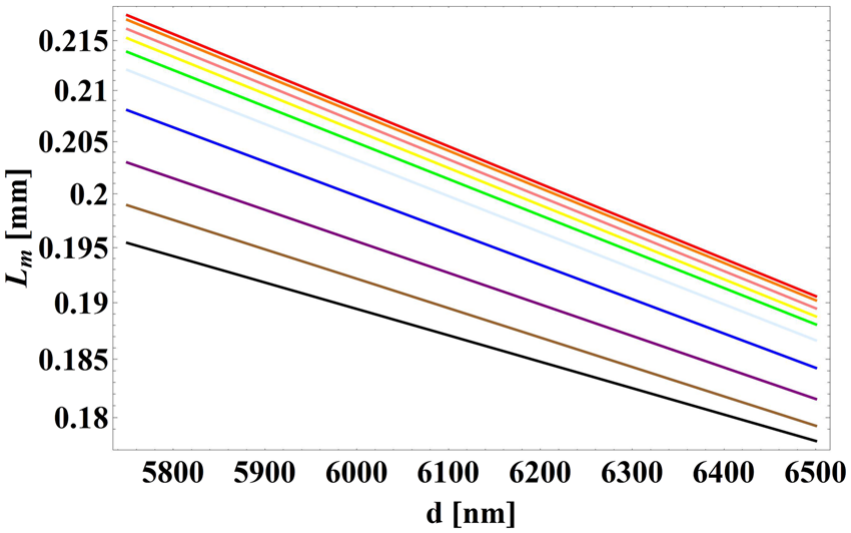
Propagation length versus waveguide’s diameter for different gap sizes for a semi-insulating InP waveguide above a glass substrate at *λ*_0_ = 20 µm. Legend is the same as Fig. 4.

Figure 3 depicts the electric field maps for a cylindrical semi-insulating InP waveguide and for a reference Si waveguide (*n*_*c*_ = 3.5) above a glass substrate (*n*_*s*_ = 1.5) at wavelength *λ*_0_ = 20 µm. Figure 4 shows the optical force exerted on the waveguides (in piconewtons per µm length of the waveguide per milliwatt input power, that is in pN · μm^−1^ mW^−1^), calculated using the above mentioned method. The negative sign of the force indicates that the force is attractive. No any guided mode exists for the waveguide diameter below 5.75 µm and 4.25 µm for semi-insulating InP and Si waveguides respectively. Figure 5 presents the propagation length of light in the semi-insulating InP waveguide calculated as

4 $${L}_{m}=\frac{1}{2{\rm{Im}}[{n}_{eff}\frac{\omega }{c}]}.$$

With smaller gap sizes, the increased field’s overlap with the lossless substrate will result in lower losses and longer propagation lengths. However, when the waveguide diameter increases, most of the field will be confined inside the waveguide and affected by losses in semi-insulating InP, resulting in shorter propagation lengths.

### SPP enhanced forces

**Figure 6:**
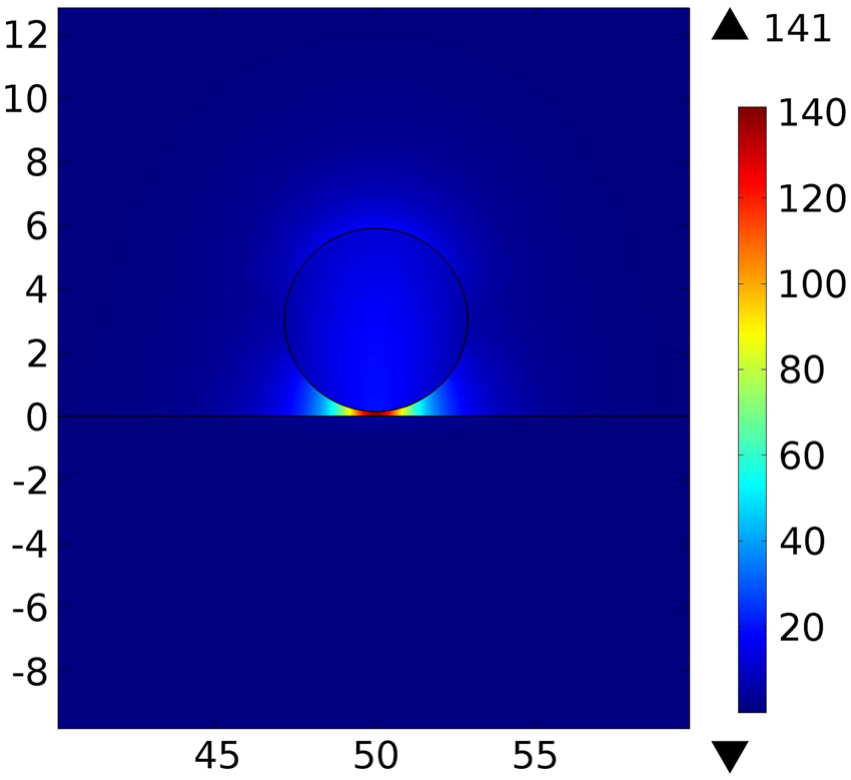
Simulated electric field [V/m] for semi-insulating InP waveguide above InP:Si substrate at *λ*_0_ = 20 µm. Spatial dimensions are in µm.

Figure 6 maps the electric field in the cross section of the semi-insulating InP waveguide above the InP:Si substrate at *λ*_0_ = 20 µm. At this wavelength, the real part of the permittivity of InP:Si is negative^12^, and TM polarized light can effectively couple to SPPs on the surface of highly doped InP:Si, if the mode effective index, *n*_*eff*_, is close to *n*_*SPP*_ given by ref. 13

5 $${n}_{SPP}={\rm{Re}}[\sqrt{\frac{{\varepsilon }_{substrate}}{{\varepsilon }_{substrate}+1}}]\,.$$

**Figure 7:**
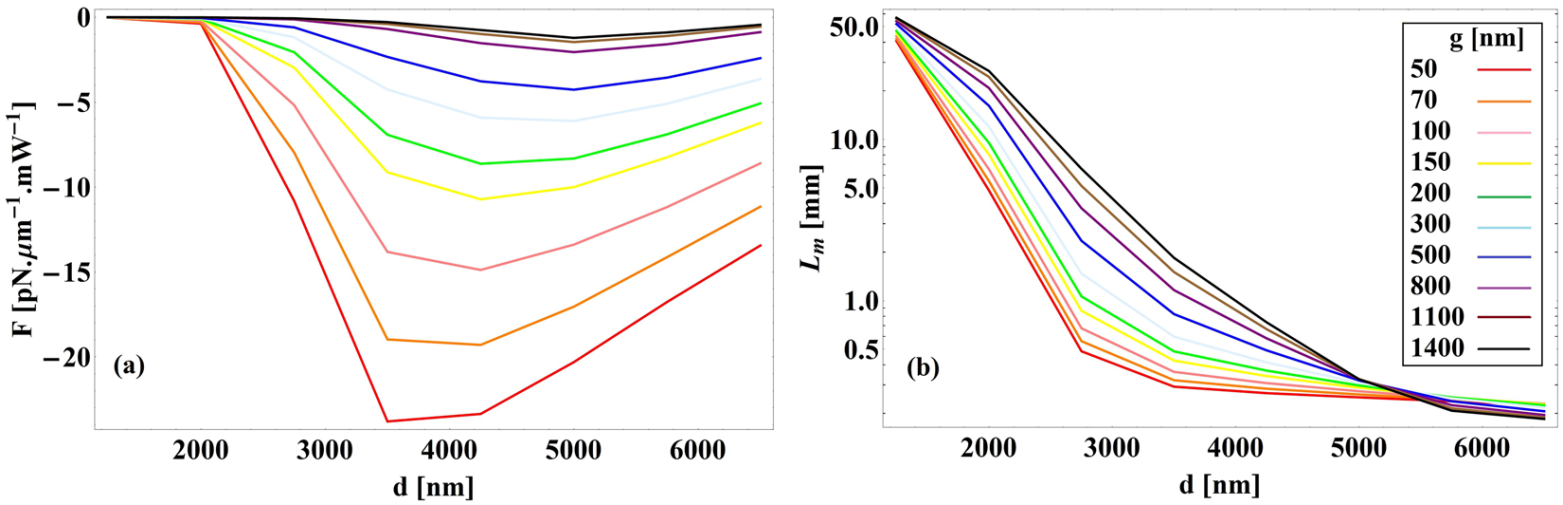
(**a**) Optical force versus waveguide’s diameter for different gap sizes for semi-insulating InP waveguide above the InP:Si substrate (**b**) propagation length at *λ*_0_ = 20 µm.

Excitation of SPPs results in hybridization of a plasmonic mode between the waveguide and the substrate and, consequently, strong confinement of the electric field inside the gap^10^. The field enhancement strongly facilitates the optomechanical interactions. Figure 7 shows the SPP enhanced forces and the propagation length for different waveguide diameters. In this case, the maximum force of −23.8 pN · μm^−1^ mW^−1^ occurs for *d* = 3.5 µm and *g* = 50 nm with the corresponding propagation length of 292.86 µm. Larger forces for the waveguide diameter *d* = 3.5 µm are a result of the maximum field confinement in the gap. As the gap size decreases, the force increases but high losses in the InP:Si substrate reduce the propagation length.

### Phonon enhanced forces

**Figure 8:**
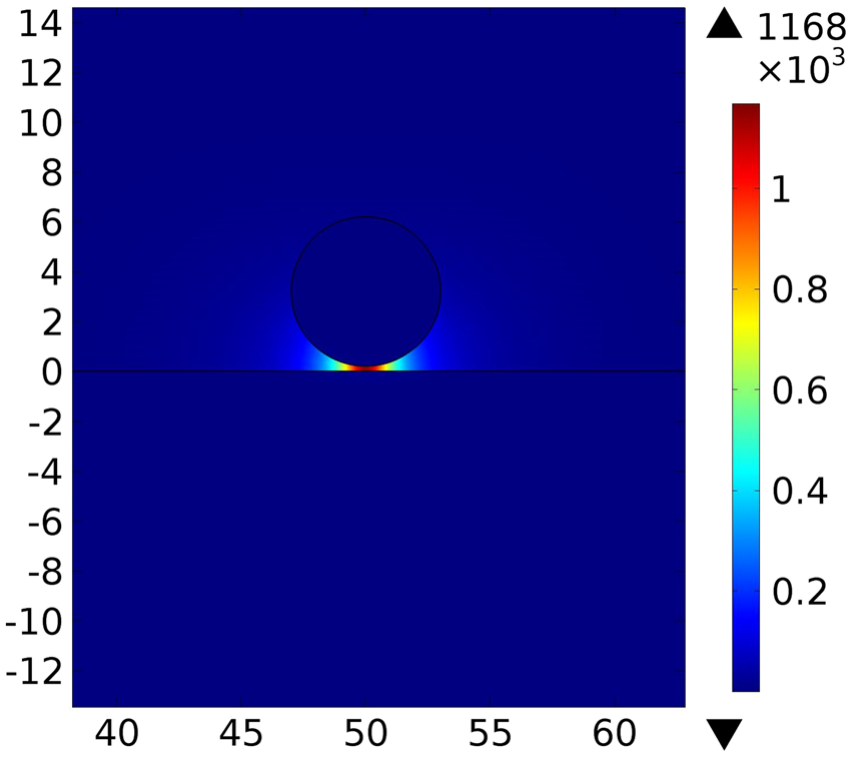
Simulated electric field [V/m] for a semi-insulating InP waveguide above the InP:Si substrate at *λ*_0_ = 32.56 µm. Spatial dimensions are in µm.

**Figure 9:**
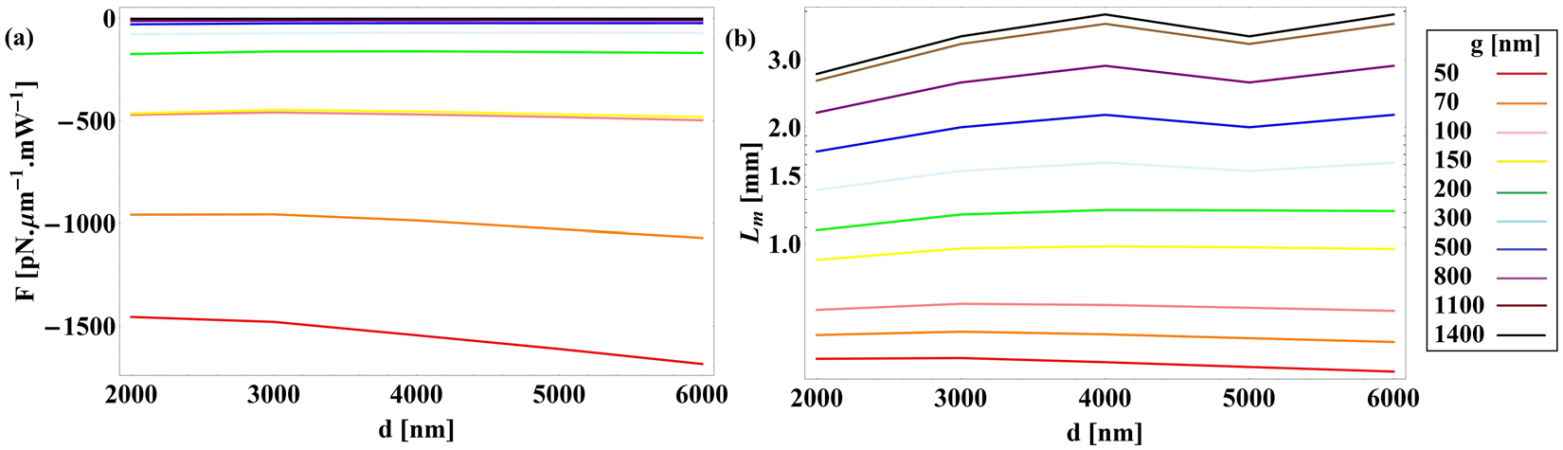
(**a**) Optical force versus waveguide’s diameter for different gap sizes for semi-insulating InP waveguide above the InP:Si substrate (**b**) propagation length of the light at *λ*_0_ = 32.56 µm.

According to Eq. (3), optical forces between the waveguide and the substrate are proportional to the spectral variations $\frac{\partial {n}_{eff}}{\partial \omega }$. InP exhibits a strong phonon absorption resonance at around 33 µm (Fig. 2) that results in acute variations of the effective mode index. This provides very large $\frac{\partial {n}_{eff}}{\partial \omega }$, which can be used to amplify optical interactions. The physical origin of this amplification is the strong enhancement of the optical near-field interaction by lattice vibrations (phonons) in InP^14^. We calculate the optical force exerted on the semi-insulating InP waveguide above the InP:Si substrate at *λ*_0_ = 32.56 µm in order to proof this concept. Figure 8 shows the simulated electric field in the cross section of the waveguide. The field is very intense in the gap due to strong concentration. Corresponding optical forces and propagation lengths are plotted in Fig. 9. The maximum force of −1685 pN · μm^−1^ mW^−1^ occurs at *d* = 6 µm and *g* = 50 nm with the propagation length of 466 µm. Such extended propagation length in this case is a consequence of lower damping in phonon polaritons in comparison to SPPs^14^ and also confinement of the field in the air gap instead of the waveguide or the substrate.

### Forces in the ENZ regime

It is intriguing to estimate optomechanical interactions in the ENZ regime, which is appealing for strongly facilitated nonlinear optical effects^15–17^. Forces exerted on electric dipole sources and polarized particles suspended above metamaterials are investigated before, and found to be repulsive in the ENZ regime^18–20^. This effect, which is similar to diamagnetic repulsion, can be used for levitation of particles which is of interest in optofluidics and low friction devices.

**Figure 10:**
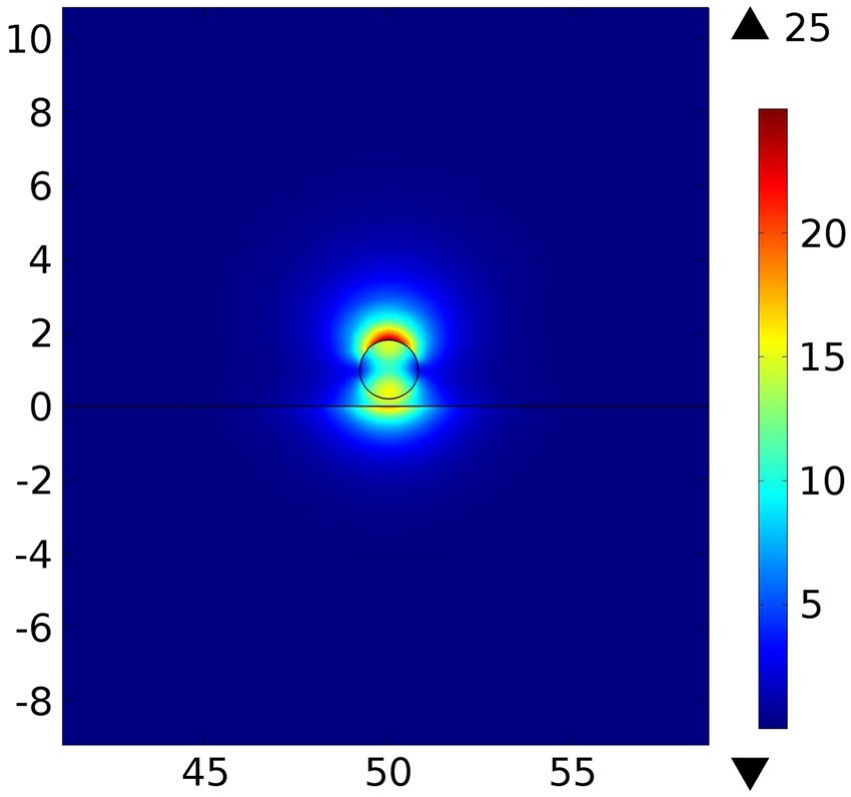
Simulated electric field norm [V/m] for semi-insulating InP waveguide above InP:Si substrate at *λ*_0_ = 6.8 µm. Spatial dimensions are in µm.

**Figure 11:**
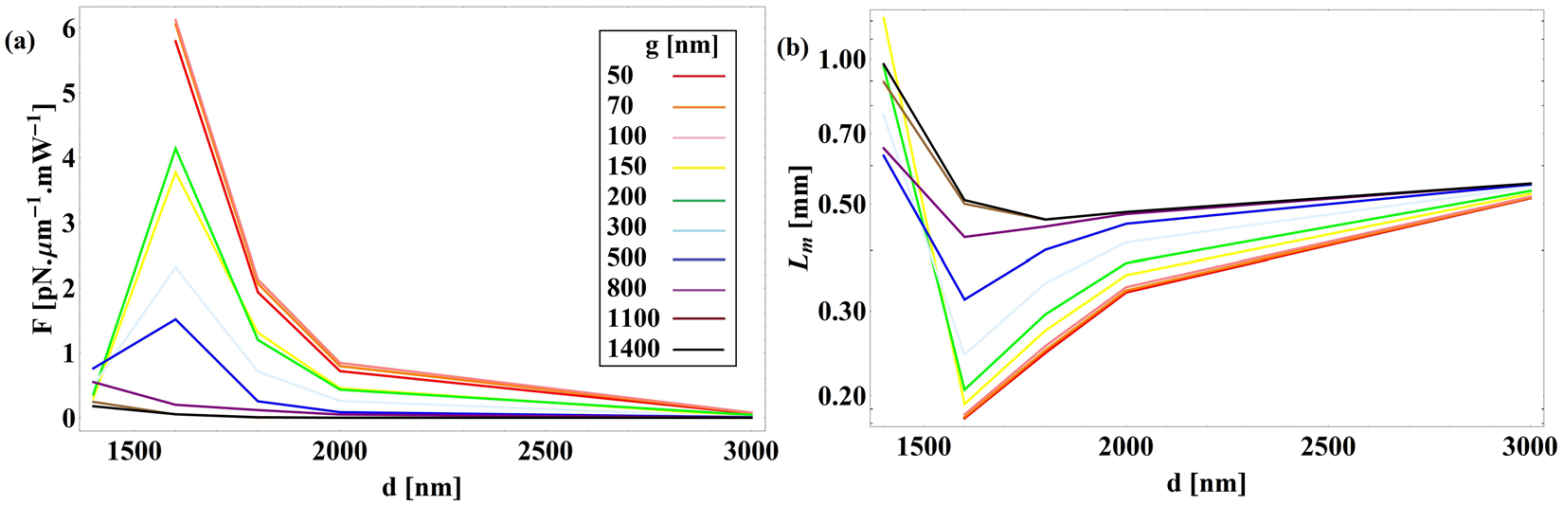
(**a**) Optical force versus waveguide’s diameter for different gap sizes for a semi-insulating InP waveguide above the InP:Si substrate (**b**) propagation length of the guided mode at *λ*_0_ = 6.8 µm.

The optical forces between a semi-insulating InP waveguide and an InP:Si substrate are calculated at *λ*_0_ = 6.8 µm, where the real part of the permittivity of the InP:Si substrate is close to zero (Re[*ε*_*substrate*_] = 0.12). Figure 10 shows the simulated electric field in the cross section of the waveguide. Figure 11 presents the force versus the waveguide diameter for different gap sizes, together with the propagation length. In this case, the resulting positive sign of the force indicates that the force is repulsive, which is a consequence of the positive sign of $\frac{\partial {n}_{eff}}{\partial g}$ in the ENZ regime. This repulsive force can be used to prevent adhesion and stiction in MEMS devices. Another interesting consequence of the ENZ regime is that the maximum force of 6.12 pN · μm^−1^ mW^−1^ happens at *g* = 100 nm, and afterwards decreasing the gap size will slightly decrease the force. In this case, unlike all of the former cases, there exists an optimum gap size which results in the maximum repulsive force between the waveguide and the substrate. No any guided mode exists at *λ*_0_ = 6.8 µm for waveguide diameters below 1600 nm, when the gap size becomes smaller than 200 nm. In the ENZ regime, InP:Si substrate has higher losses than the semi-insulating InP waveguide which results in lower propagation length as the gap size decreases.

## Discussion and Conclusion

Optically induced forces in waveguides, which arise due to the coupling between the evanescent tail of a guided mode and a substrate or another waveguide, can be exploited to actuate MEMS devices and optically tunable photonic devices. InP-based waveguides are investigated here as a proof of concept. InP which is a CMOS compatible material, can be epitaxially grown and processed in order to fabricate suspended waveguides that benefit from different optical interaction enhancement processes at different wavelengths. Simulations show that upon coupling to SPPs (at *λ*_0_ = 20 µm), the attractive force exerted on a semi-insulating InP waveguide lying above a highly doped InP:Si substrate increases by an order of magnitude in comparison to the waveguide above the dielectric substrate, which is a result of the amplified electric field below the waveguide^10^. In the phonon absorption regime (at *λ*_0_ = 32.56 µm) the force is about three orders of magnitude higher than that for the waveguide above the dielectric substrate, whereas the propagation length also increases by a factor of two. Longer propagation length, in comparison to the waveguide above the dielectric substrate, is a result of the confinement of the field in the airgap instead of the waveguide or the substrate, and also relatively weaker damping of phonon polaritons in comparison to the SPPs^14^. The force in the ENZ regime (at *λ*_0_ = 6.8 µm) was observed to be repulsive and larger than the force for the waveguide above the dielectric substrae. This repulsive force can be applied to prevent stiction of the micro-cantilever to the substrate. Unlike the SPP and the phonon excitation regimes in which the force always increases by decreasing the gap size, in the ENZ regime there exists the optimum gap size where the force is maximal. The propagation length of the waveguide mode in all above mentioned cases is greater than 15 times the input light wavelength which is a result of low losses in InP:Si.

Transverse deflection in the middle of a fixed-fixed beam under a line force *F* is given by

6 $$w=\frac{F{(\frac{L}{2})}^{4}}{24EI}$$

where *L* is the length of the beam, *E* is the Young modulus of the beam’s material which is equal to 71 GPa for InP^21^, and *I* is the second moment of area for the beam’s cross section which is given by $\frac{\pi }{4}{(\frac{d}{2})}^{4}$ for a circular cross section. Using the above equation and the forces found in the previous sections, the maximum static deflection of a waveguide with parameters *d* = 3.5 µm and *L* = 200 µm are found to be equal to 0.19 and 12.08 nm/mW for the SPP enhanced case and the phonon enhanced case respectively. Deflection will be much larger for a waveguide with a rectangular cross section (considering the width and the height of the rectangular cross section equal to *d* and *d*/3) due to the larger effective mode area under the waveguide and also the smaller second moment of area of the rectangular cross section. In addition, applying a periodic force using a pulsed input with a frequency close to one of the natural mechanical frequencies of the waveguide will drastically increase the deflection amplitude. Taking advantage of the tunability of the ENZ and SPP regimes by adjusting the free carrier concentration (plasma wavelengths from 7.4 to 22.7 µm reported for InP:Si with different carrier concentrations in ref. 12), the huge optical forces can be regarded as a novel actuation method for future of the MEMS devices.
